# Editor’s Book Table

**Published:** 1870-04

**Authors:** 


### Editor's Book Table.

[NOTE.—All works reviewed in the columns of the CHICAGO MEDICAL JOURNAL may be found in the extensive stock of W. B. Keen and Cooke, whose catalogue of medical books will be sent to any address upon request.]

*The Physiology of Man; Designed to Represent the Existing State of Physiological Science, as Applied to the Functions of the Human Body.* By AUSTIN FLINT, JR., M.D., Professor of Physiology and Microscopy in the Bellevue Hospital Medical College, etc., etc. Secretion; Excretion; Ductless Glands; Nutrition; Animal Heat; Movements; Voice and Speech. New York: D. Appleton & Company, 90, 92, and 94 Grand Street. 1870. Pp. 526.

THIS is the third volume of a series commenced several years since by the younger Flint. The first volume, it will be recollected, discussed the Blood, Circulation and Respiration—the second, Alimentation, Digestion and Absorption. A concluding volume will be devoted to the functions of the nervous system, and the process of generation and development. Each volume, however, constitutes a separate and distinct treatise, being complete in itself.

The previous volumes have been well received by the profession, and the author is well known — beyond the necessity of an introduction. The present volume will enhance his reputation as a pains-taking, careful student and observer. Aside from numerous experiments in verification of the reports of others, he has instituted many original experiments, and, particularly with reference to the excretory function of the liver, has developed new facts of great interest.

A feature in the work is the effort "to draw as closely as possible, the line of distinction between secretions proper and excretions," and he believes the information in our possession is now of so positive a character, that we are able to subject the processes to definite generalization.

Personally, however, he indulges very little in attempts to generalize, an omission which may well be pardoned since he everywhere avoids any mere speculation or rhetoric. So great is his apparent dread of premature generalization that there is, if anything, a little too much grouping of facts, with too little effort to point out their essential relations. This is illustrated, partially, by the cavalier method in which he dismisses the doctrines of Dr. Beale, but more particularly by his discussion of the relation of the nervous system to the process of secretion. In his subsequent volume we hope to see this relation pointed out in a clearer and more philosophic manner. The limited and incorrect generalizations of Hall are scarely improved upon by admission of the still more restricted system of Dr. Campbell.

But we are in no mood to find fault with a work which, taken as a whole, is so thoroughly satisfactory, and which does honor to American medical literature.

Those who have the first two volumes will, of course, buy this, and those who have not should at once buy the three.

The publishers have brought out this volume in uniform with the preceding volumes, and in a style which reflects great credit upon them.

*The Cell Doctrine; Its History and Present State. For the use of Students in Medicine and Dentistry. Also a Copious Bibliography of the Subject.* By JAMES TYSON, M.D., Lecturer on Microscopy in the University of Pennsylvania, etc., etc. With a Colored Plate and other Illustrations. Philadelphia: Lindsay & Blakiston. 1870. Pp. 150. $2.00.

THIS treatise is brought out in the same style with the London edition of Dr. Beale's essay on Protoplasm. It is perspicuously written, and gives concise statements of the views which have been entertained with reference to the vegetable and animal cell from first discovery to the present time. Especial attention is given to the doctrines of Schleiden and Schwann, Henle, Martin Barry, Prof. Goodsir, Huxley (1853), J. Hughes Bennett, Todd and Bowman, Virchow, Dujardin, Schultze, Beale, Robin and Huxley (1869).

The author adopts, with some modifications, the views of Dr. Beale. We thank him, on our own part, for the Bibliography he has given us, which is alone worth to us more than the price of the book.

We advise all interested in Physiology to give this essay a careful reading.

*Modern Therapeutics; A Compendium of Recent Formulæ and Specific Therapeutical Directions.* By George H. Napheys, A.M., M.D., Chief of Medical Clinic of Jefferson Medical College, etc., etc. Philadelphia: S. W. Butler, M.D., 115 South Seventh Street. 1870. Pp. 390.

Something like a couple of hundred professional celebrities are represented in this compend. What they say about particular methods in therapeutics, and their formularized notions are herein, more or less, set forth. The formulæ are arranged in classes under the general variety of affections for which they are presumed, by authority, to have been found useful. For the busy practitioner it will be found very convenient to have at hand something of this sort to suggest methods with which he has previously been unfamiliar. It seems hardly necessary, in this age, to caution against the tendency to depend upon routine treatment which books of the kind are likely to foster.

The contemporaneous use of a little good-natured skepticism will avoid this result, and meanwhile the book will be a good floater for those who cannot swim, and an occasional relief for those who can.

The delay in issue (see advertising pages of Journal) has been well made up to buyers by the addition of a hundred extra pages without increase of the proposed price.

*Health by Good Living.* By W. W. Hall, M.D., Editor of Hall's Journal of Health, etc., etc. New York: Hurd & Houghton. Cambridge: Riverside Press. 1870. Pp. 277.

Written for popular perusal, it is one of the few books of the kind we can conscientiously recommend. To our professional brethren who are afraid of "a good square meal," we especially commend it. It is full of good sense and is professionally unexceptionable in tone.

### Pamphlets.

*Second Annual Report of the New York Orthopædic Infirmary.* Located at 1299 Broadway.

*Address before the St. Clair and Sanilac County (Michigan) Medical Society.* By the retiring President, J. T. Travers, M.R.C.S.L.; with reports of committees, etc., etc.

In the same pamphlet we notice a highly interesting case, reported by our highly esteemed friend, Surgeon (U. S. A.) M. K. Taylor, of poisoning by the biniodide of mercury. Dr. T. points out the fact, that, unlike the bichloride, the biniodide forms no precipitate with albumen, which although recommended in the books as antidotal, is clearly not so, and from its preventing the action of other remedies should not be given. He is satisfied that in poisonous doses the biniodide is sedative and stupefying, and only the most prompt and efficient efforts will avail in saving life. He observes:

"So far as my means of judging indicate, the employment of liquor potassa, solutions of carb. potassa, extemporaneously prepared, lime-water followed immediately by bicarb. potassa; and the sulphide of ammonia, or potassa, the latter being the better, followed by emetics, or the stomach-pump, constitute our chief antidotes."

The very general use of this powerful agent as an antisyphilitic renders farther investigations of its best antidote desirable.

*Historical Letter on the Introduction of Anæsthetics in Dentistry and Surgery in America, and on their First Employment in Midwifery in Great Britain.* By Sir J. Y. Simpson, Bart., M.D., D.C.L., Professor, etc. Edinburgh.

A spicy reply to the elder Bigelow, of Boston (Massachusetts, U. S. A.). The eminent Scotchman, herein, shows that, for at least once, Boston is not entitled to *all* the honors.

*Report of the Board of Managers, Superintendent and Treasurer of the Western Lunatic Asylum of Kentucky, for the year* 1869.

*Anniversary Oration Delivered before the Medical Society of the District of Columbia*, by J. M. TONER, M.D.

THIS address gives a complete historical sketch of the Society from its organization to the present time. It also includes a history of the military hospitals during the war, museums, etc., etc. As is the case with all things undertaken by this most estimable professional gentleman, it is well done, and will form a permanent chapter in the medical history of the country.

*Michigan University Medical Journal.* March, 1870. Conducted by the Faculty of the Medical Department. R. A. Beal, Ann Arbor, Mich., publisher. $3.00 a year.

THIS is the initial number of a new medical monthly. It is neatly printed. Besides the editorial conduct of the Faculty, its issue is facilitated by a board of four "managing editors." If the publisher will now insist upon "payment invariably in advance," it will go far toward the achievement of success. The bane of medical journalism is the credit system, as the present writer is willing to make solemn affidavit. If the Michigan publisher don't believe this we offer him several thousand dollars of arrearages to this JOURNAL before it adopted the cash system. They will be sold at an enormous sacrifice in view of the approaching resumption of specie payments.

*On the Influence of Mental Activity on the Excretion of Phosphoric Acid by the Kidneys. Silliman Prize Thesis.* By LUTHER HODGES WOOD, Ph.B., M.D.

THIS paper recounts a series of experiments instituted for the purpose of confirming or refuting the propositions of Dr. T. R. Noyes (Am. Jour. Med. Sci., Oct., 1867) that the urine of the day is uniformly alkaline, and that of the night as uniformly acid. Dr. Noyes suggested, as accounting for this fact, that the causes of acidity were operating in both periods, and that the great increase of the alkaline phosphates in the daytime overbalanced the acid reaction thus produced. The results given by Dr. Wood's experiments are tabulated, and also illustrated, by a series of nine diagrams.

The results are summed up as follows:

1. The *amount of urine* excreted varies at different periods of the day, even on a fixed diet; the day-urine exceeds the night-urine in the ratio of 3 to 2. The largest amount is excreted during the forenoon, the next largest in the afternoon, then comes that of the latter part of the night, and lastly that passed in the early part of the night.

2. The *density* of the urine varies inversely as the amount of urine passed; the morning-urine having a higher specific gravity than that excreted at night.

3. The *total amount of solids* excreted is greater during the day than during the night by nearly 50 per cent.; thus showing that the density is not diminished in proportion to the amount of urine passed.

4. The *reaction* of the day-urine is uniformly alkaline, that of the night-urine acid; while, however, acid urine is excreted during both periods of the night, it is the morning-urine only that is alkaline, that of the afternoon being acid.

5. The total *phosphoric acid* excreted per hour on an ordinary diet, is largest during the day, rising highest after the principal meal; while on a fixed diet, the excretion is greatest at night, the maximum being reached dnring the first half of the night, the amount diminishing in the afternoon; it is less still at 7 A.M. and least at 1 P.M.

6. The *alkaline phosphates*, when an ordinary diet is taken, are greater by day than by night; on a fixed diet the reverse is true.

7. The *earthy phosphates*, on the other hand, are largest in amount during the day, both on ordinary and fixed diets.

8. The *total phosphoric acid* is very greatly affected by the amount and kind of food taken.

9. The *variations* in the amount of phosphoric acid, considered as a whole, are not sufficient to afford any indication of the previous mental condition.

10. The *alkaline phosphates* are only *slightly increased* on increasing the amount of mental labor.

11. The *earthy* are *diminished* under the same conditions, by an amount varying from 20 to 40 per cent.

12. *No such increase of phosphoric acid* as would be required by the theory of the disintegration of nervous tissue during action, was observed in these experiments.

13. The *alkalinity* of the day-urine is not due to the presence of alkaline phosphates in excess.

*Relaxation of the Pelvic Symphyses during Pregnancy and Parturition.* By FREDERICK G. SNELLING, M.D. Published by W. A. Townsend & Adams, New York.

PREMISING that this condition has been known and commented on since the time of Hippocrates, and yet the allusions to it in the systematic treatises are scant and rare, he continues;

"The affection appears to consist of a relaxation of the pelvic articulations, becoming apparent suddenly after parturition, or gradually during pregnancy; and permitting of a degree of mobility of the pelvic bones which effectually hinders locomotion, and gives rise to the most peculiar, distressing, and alarming sensations. It can, perhaps, be best illustrated by the following case by Dr. Duplain.

"The patient, Madame ——, was 26 years of age, of a lymphatic temperament, married four years, and the mother of three children. The last child was born about the middle of May, 1867, after a labor lasting twenty-two hours, and was a child of unusual size. After its birth she was almost constantly confined to the bed, from the difficulty, and indeed impossibility of walking, and a singular and distressing sensation, as if the abdominal viscera were about to fall through the pelvic outlet. She also had vague pains, increased on motion, in the hips, at the symphysis pubis, and in the loins. As for the other symptoms, her appetite was good, her sleep sound, pulse normal, bowels regular, and urinary secretions healthy. The vaginal touch disclosed no malposition, or other disturbance of the uterine system. On palpation, the abdomen was supple and lax. On examining the patient in a recumbent position, the lower limbs presented nothing abnormal; their sensibility was intact, and movement was free and painless. But immediately upon arising, the sensation complained of returned with much severity, walking was accomplished with difficulty, and she dragged one foot after the other, inclining herself to the right and left as the case might be. On compressing the pubic and sacro iliac symphysis some pain was experienced.

"From the symptoms supervening upon delivery, the physician, M. Duplain (eliminating the possibilities of diseases of the spinal cord, of the pelvic viscera, lumbo-abdominal neuralgia, etc.,) judged it to arise from a relaxation of the pelvic symphyses, and the sequel justified the accuracy of the diagnosis.

"A bandage was placed about the pelvis and hips in such a manner as to compress and confine the articulations firmly. Walking immediately became easy: she could maintain an upright position, the pains disappeared, and at the end of two months, without any other treatment, the patient left off her bandage and found herself entirely cured.

"This may be regarded as a typical case of *uncomplicated* relaxation of the pelvic symphyses."

A number of other cases are given with cuts illustrative. Suppurative inflammation of the symphyses sometimes occurs, with exhausting discharge, rupture, caries, etc. For the diagnosis little instruction is needed, provided the physician remembers the possibilities of the case. The treatment involves that for the prevention or removal of inflammation and after its subsidence the wearing of a pelvic bandage for a lengthened period.

Profs. Fordyce Barker and Isaac E. Taylor, remarked, with reference to this paper, that it recalled attention to a subject of great importance. Each had seen several cases, and they endorse the use of the bandage, although Prof. Meigs thought it of little service.

It is clear from reading this paper that the supposed rarity of the lesion is not so great but that the subject demands the attention of practitioners.

*Half-Yearly Compendium of Medical Science.* Part V, January, 1870, Pp. 304, has just come to hand. From such examination as we have had time to give it, the present volume is an improvement on its predecessors, the editors — Drs. Butler, Brinton and Napheys — having devoted careful attention to the character of selections. $3 a year. Single numbers, $2. Nos. 1 to 4, inclusive, $5. Address — S. W. Butler, M.D., 115 South Seventh Street, Philadelphia.

### Literary.

*Mauprat.* A Novel. By George Sand. Translated from the French by Virginia Vaughan. Boston: Roberts Brothers. 1870.

The publishers are doing the American public good service by introducing to its readers the works of a most remarkable woman; a woman whose name has been widely pronounced, but whose works have been read in this country to an extent vastly below their real merit.

*A Day by the Fire;* and other Papers hitherto uncollected. By Leigh Hunt. "Matchless as a fireside companion."—*Elia.* Boston: Roberts Brothers. 1870. Poetic prose from one of our most genial writers.

*Red as a Rose is She*, is the quaint title of a novel which we have not had time to look over, but being published by the Appletons and sold by W. B. Keen & Cooke, is of course good.

